# Lysine 222 in PPAR γ1 functions as the key site of MuRF2-mediated ubiquitination modification

**DOI:** 10.1038/s41598-023-28905-5

**Published:** 2023-02-03

**Authors:** Yucheng Fan, Fangjing Xu, Rui Wang, Jun He

**Affiliations:** 1 Department of Pathology, The First People’s Hospital of Shizuishan, Affiliated to Ningxia Medical University, Shizuishan, China; 2 School of Clinical Medicine, Ningxia Medical University, Yinchuan, China; 3 School of Basic Medical Sciences , Ningxia Medical University, Yinchuan, China; 4 Department of Cardiovascular Internal Medicine, General Hospital of Ningxia Medical University, Yinchuan, China

**Keywords:** Molecular biology, Medical research

## Abstract

Peroxisome proliferator-activated receptor gamma (PPAR γ) plays key roles in the development, physiology, reproduction, and homeostasis of organisms. Its expression and activity are regulated by various posttranslational modifications. We previously reported that E3 ubiquitin ligase muscle ring finger protein 2 (MuRF2) inhibits cardiac PPAR γ1 protein level and activity, eventually protects heart from diabetic cardiomyopathy; furthermore, by GST-pulldown assay, we found that MuRF2 modifies PPAR γ1 via poly-ubiquitination and accelerates PPAR γ1 proteasomal degradation. However, the key ubiquitination site on PPAR γ that MuRF2 targets for remains unclear. In the present study, we demonstrate that lysine site 222 is the receptor of MuRF2-mediated PPAR γ1 ubiquitination modification, using prediction of computational models, immunoprecipitation, ubiquitination assays, cycloheximide chasing assay and RT-qPCR. Our findings elucidated the underlying details of MuRF2 prevents heart from diabetic cardiomyopathy through the PPAR γ1 regulatory pathway.

## Introduction

Peroxisome proliferator-activated receptor gamma (PPAR γ) is a member of the nuclear hormone receptor superfamily transcription factors and is a master regulator of adipogenesis, glucose homeostasis, and cell growth^1–3^. In mouse, there are two isoforms of PPAR γ: PPAR γ1 and PPAR γ2; the sequences are identical in both isoforms except 30 extra amino acids are contained in the NH2-terminus of PPAR γ2^1,4^. Under normal physiological conditions, PPAR γ2 is only observed in adipocytes and is absolutely required for adipogenesis^1,5,6^. PPAR γ1 is expressed universally in many cell types, such as cardiomyocytes, nerve cells, monocytes/macrophages, T lymphocytes, vascular endothelial cells, smooth muscle cells, breast, and colonic epithelium, and exerts biological functions on cardiovascular, nervous, immune, and gastrointestinal systems^1,7–11^. The roles of PPAR γ in cardiovascular disorders have been discussed recently. It was reported that PPAR γ protects against IL-1β-mediated endothelial dysfunction through a reduction of oxidative stress responses in high-fat diet-fed apolipoprotein E-deficient mice^12^. PPAR γ ligands rosiglitazone and 15-deoxy-Δ^12,14^-prostaglandin J2 showed suppressive effects on angiotensin II-induced cardiac fibrosis in rat^13^. The expression of PPAR γ1-regulated target genes involved in fatty acid metabolism was increased and triglyceride was accumulated in high-fat diet induced hypertrophic hearts^14^. Studies have shown the function of PPAR γ is directed by its activity or expression level. Downregulation of the PPAR γ gene resulted in a ventricular membranous septation defect of the embryonic heart at E14.5^15^. Enhances of the occupancy of PPAR γ on the promoters of critical fatty acid transporters led to increased fatty acid uptake and lipid accumulation in murine hearts^16^. The insulin resistance and cardiac dysfunction of diabetic cardiomyopathy mice can be ameliorated by decreasing the abundance of PPAR γ1 protein^14^. Therefore, identifying the factors that regulate PPAR γ activity and expression is essential for understanding its intrinsic mechanism for regulating heart function, subsequently beneficial for intervention of cardiovascular diseases. PPAR γ is subjected to posttranslational modifications, such as phosphorylation, acetylation, methylation, and sumoylation and ubiquitination. Previously, in cell-based in vitro study, we demonstrated that E3 ubiquitin ligase muscle ring finger protein 2 (MuRF2) modifies PPAR γ1 by poly-ubiquitination and promotes its degradation through the proteasomal pathway, therefore inhibits PPAR γ1 activity^14^. However, the specific ubiquitination site(s) which MuRF2 ligates ubiquitin and PPAR γ1 has not yet been described at all. The present study aims to identify the ubiquitination site(s) in human PPAR γ1 by computational prediction tools, ubiquitination and cycloheximide chasing assays, and RT-qPCR experiments. Our findings add detail to the mechanism that MuRF2 prevents heart from diabetic cardiomyopathy through the PPAR γ1 regulatory pathway.

## Results

### Screening of the candidate ubiquitination sites

**Figure 1:**
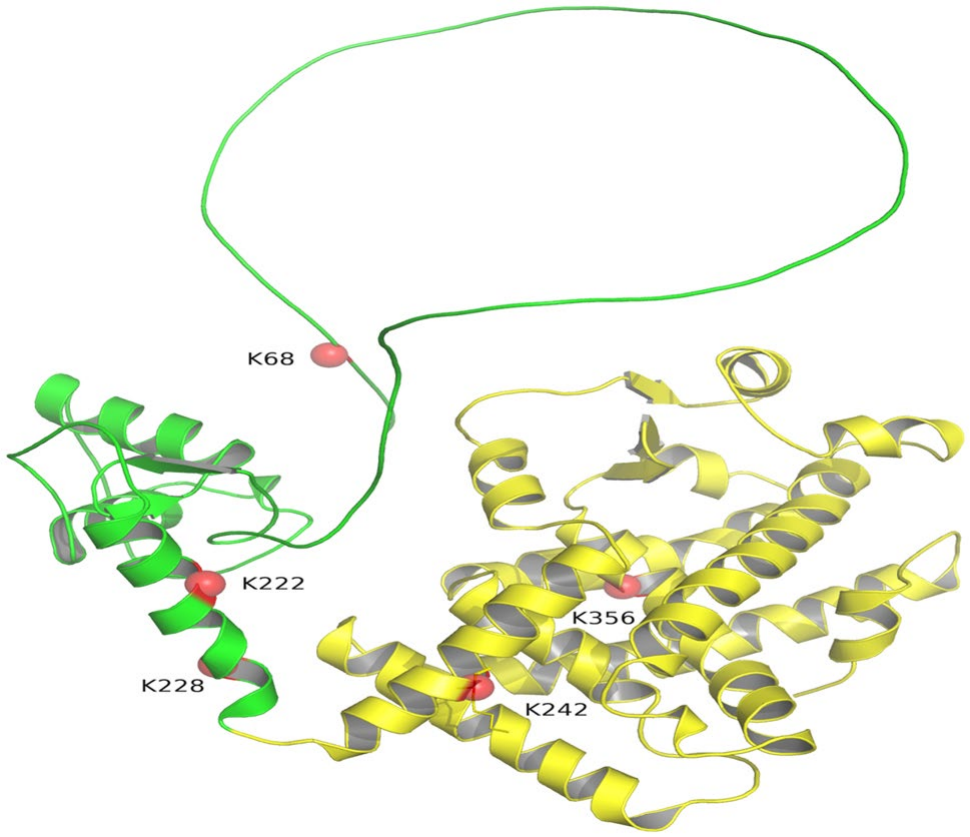
Distribution of the candidate sites in PPAR γ1 protein. The spatial model of PPAR γ1 molecule was derived by PyMOL software (version 2.4.0 openvr; DeLano Scientific, San Carlos, CA, USA; https://pymol.org/2/). The filtered ubiquitination sites K68, K222, K228, and K242 and K356 were showed as red spheres.

PPAR γ is a lysine-rich protein. To identify the amino acid residue(s) targeted by MuRF2, we first characterized the specific ubiquitination site(s) on human PPAR γ1 by computational prediction tools, UbiSite and UbiProber, based on their acceptable sensitivity as well as specificity. Site with a SVM score > 0.8 in UbiProber or a SVM score > 0.9 in UbiSite was regarded as a candidate. According to the criteria, the positions on the amino acid sequence of PPAR γ1 K68, K222, K228, and K242 and K356 were screened out (Table 1). The candidate sites were visualized in a 3D PPAR γ1 molecular model established by PyMOL software (Fig. 1), PyMOL software (version 2.4.0 openvr; DeLano Scientific, San Carlos, CA, USA; https://pymol.org/2/) was used to view the graphic.

**Table 1:** Prediction of the lysine residues in PPAR γ1 by computational tools.

Gene names	Ub sites	SVM core	Forecast tool	Nucleotide sequence	Modified sequence
PPARG(NM_138711)	K68	0.6161	UbiSite	CTG**AAA**CTT	CTG**AGA**CTT
		0.8845	UbiProber		
PPARG(NM_138711)	K222	0.9409	UbiSite	ATA**AAG**TCC	ATA**AGG**TCC
		0.8319	UbiProber		
PPARG(NM_138711)	K228	0.9024	UbiSite	ACC**AAA**GCA	ACC**AGA**GCA
PPARG(NM_138711)	K242	0.8380	UbiSite	GAC**AAA**TCA	GAC**AGA**TCA
		0.866	UbiProber		
PPARG(NM_138711)	K356	0.8502	UbiSite	CGA**AAG**CCT	CGA**AGG**CCT
		0.8179	UbiProber		

Based on the ubiquitin conjugation web resources UbiProber and UbiSite combined using the SVM score, the residues K68, K222, K228, and K242 and K356 in PPAR γ1 were screened out and regarded as the candidate ubiquitination sites (original tables are presented in Supplementary Tables S1, S2).

### In vitro ubiquitination by GST-pulldown assay

**Figure 2:**
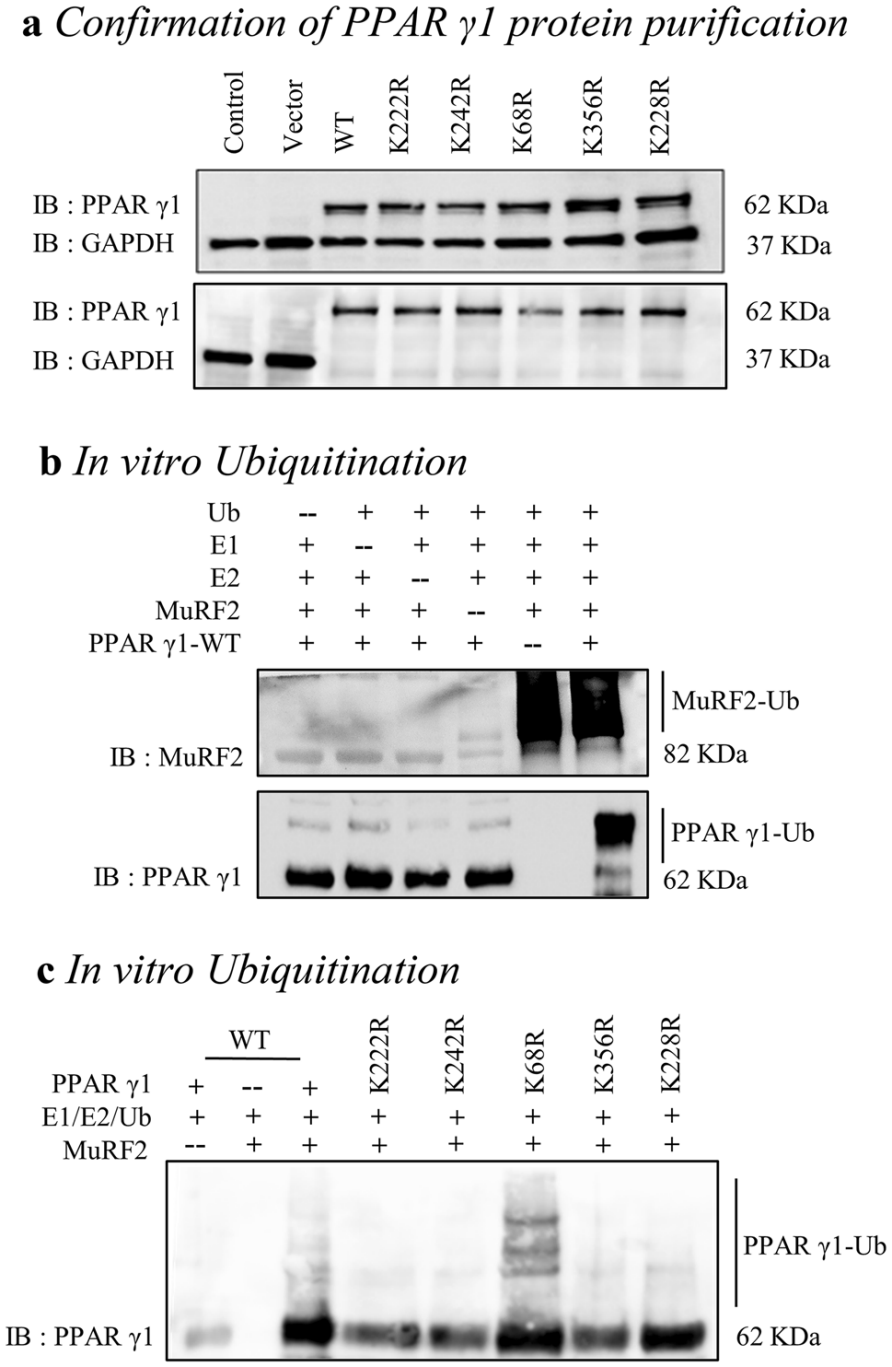
Identification of ubiquitination site(s) in vitro by GST-pulldown assay (original blots are presented in Supplementary Figs. S1–S3). (**a**) The upper: the over-expression plasmid of PPAR γ1 or the mutant of PPAR γ1 (K68R, K222R, K228R, K242R, K356R) was transfected in HEK293T cells respectively. The PPAR γ1 proteins levels were verified by immunoblot, and no endogenous PPAR γ1 was observed in 293T cells. The lower: immunoblot analysis of the purification efficiency of PPAR γ1 protein. Compared to the negative and positive controls, no GAPDH protein was detected in the total protein derived from the PPAR γ1 and mutants transfected groups, indicating that purified proteins of PPAR γ1 and the mutants were obtained. (**b**) The upper: MuRF2 auto-ubiquitination was demonstrated by immunoblot of MuRF2 (lane 5 and lane 6). The lower: MuRF2’s ability to ubiquitinate PPAR γ1. The smeared PPAR γ1 was observed obviously in the full reaction (the far right lane 6). (**c**) Residue K68 showed the remote possibility of being the ubiquitination site. Except the PPAR γ1 K68R, all the protein stability of the mutant PPAR γ1 K222, K228, and K242 and K356 were weakened in the presence of MuRF2 compared to that of the PPAR γ1 protein, indicating the dispensability of lysine site K68 in MuRF2 mediated PPAR γ1 ubiquitination modification. Ub, ubiquitin; E1, ubiquitin activating enzyme; E2, ubiquitin conjugating enzyme.

To test the function of these ubiquitination sites, the lysine (AAA) was mutated to arginine (AGA, AGG), and the mutant residues were marked as K68R, K222R, K228R, and K242R and K356R. Next, the plasmids of PPAR γ1 or the mutant PPAR γ1 (K68R, K222R, K228R, K242R, K356R) were cotransfected in HEK293T cells respectively; then total protein was extracted and purified using anti-GFP affinity beads 4FF. The purification of PPAR γ1 protein was confirmed by immunoblot (Fig. 2a, the lower). Finally, the in vitro ubiquitination reaction system was constructed and assayed. We found that PPAR γ1 was poly-ubiquitinated by MuRF2 (Fig. 2b, the far right lane of the lower), and interestingly, MuRF2 auto-ubiquitination was observed in this case (Fig. 2b, the upper) as we previously reported^14^. As shown in Fig. 2c, the ubiquitination levels of PPAR γ1 K222R and K242 mutants (lane 4 and lane 5) were significantly decreased, the levels of K68R, K228R and K356R mutants (lane 6, lane 7, lane 8) were not remarkably reduced compared to that of the PPAR γ1 (lane 3). The data implied that K68, K228 and K356 are less likely the target for MuRF2 ubiquitinating PPAR γ1.

### In vivo ubiquitination by cell transfection study

**Figure 3:**
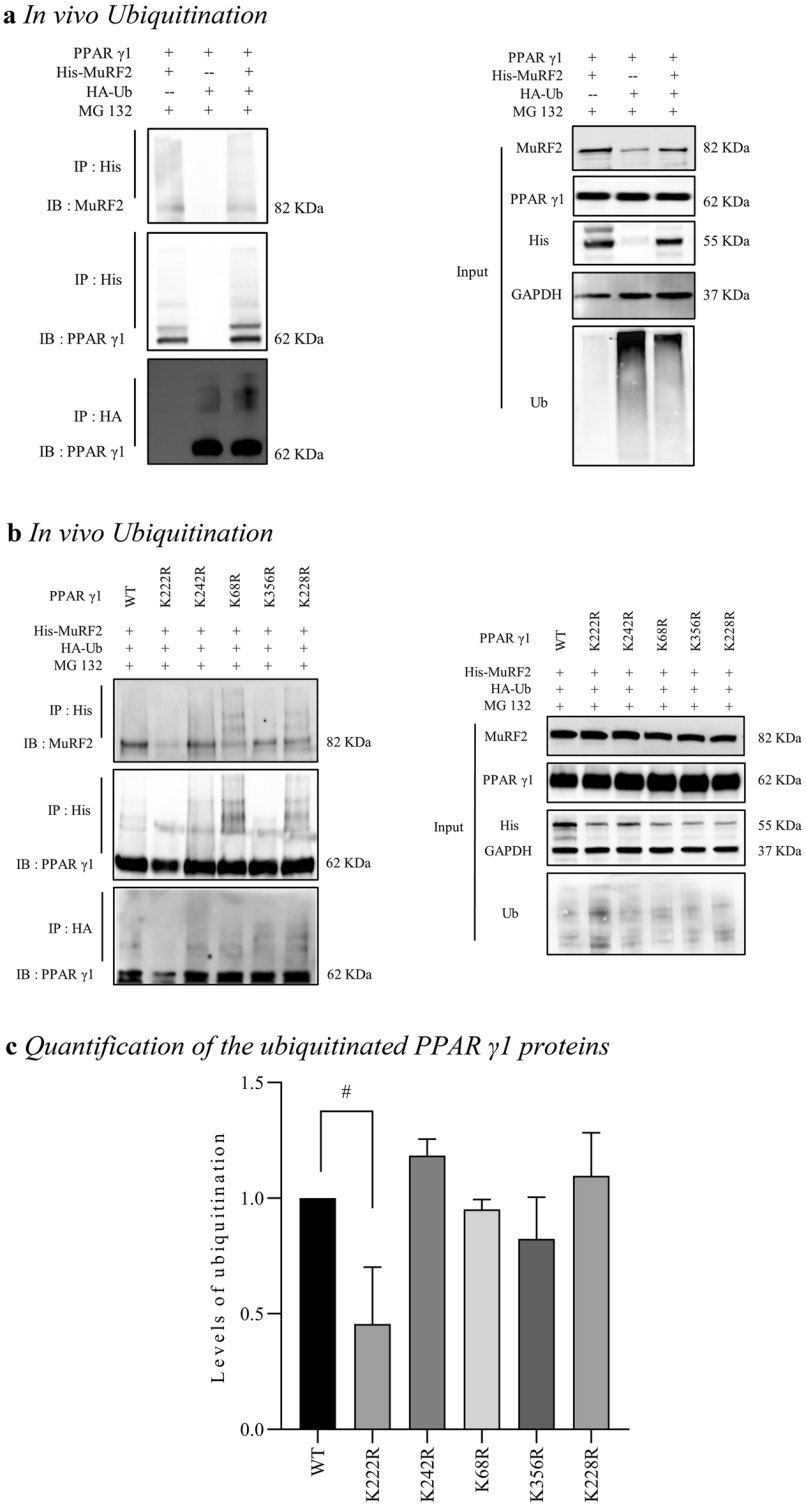
Identification of ubiquitination site(s) in vivo by transfection study (original blots are presented in Supplementary Figs. S3–S5). (**a**) MuRF2 interacted with PPAR γ1 and modified PPAR γ1 via poly-ubiquitination. HEK 293T cells were co-transfected with plasmids His-MuRF2, HA-Ub and PPAR γ1. Cells were treated with proteasome inhibitor MG132 for 6 h before harvest a, and followed by immunoprecipitation and immunoblot analyses. The immunoprecipitation studies identified the interaction between MuRF2 and PPAR γ1 proteins, and the immunoblots of PPAR γ1 indicated that MuRF2 modified PPAR γ1 protein by poly-ubiquitination (the right lanes of the middle and the bottom). (**b**) Lysine site 222 exhibited the essential for MuRF2 ubiquitinating PPAR γ1. HEK 293T cells were co-transfected with plasmid of His-MuRF2, HA-Ub and PPAR γ1 wild type or mutant K68R, K222R, K228R, K242R and K356R respectively. The immunoblots of PPAR γ1 demonstrated the ubiquitination level of K222R mutant was significantly reduced (the middle and the bottom of the left figure), indicating residue K222 on PPAR γ1 is the target for MuRF2. (**c**) Compared to the ubiquitination level of the wildtype PPAR γ1, the level of the mutant K222R was decreased significantly (*p* < 0.05). No difference was observed between the wildtype and the other mutants.

We performed immunoprecipitation studies by cotransfecting HEK293T cells with plasmids His-MuRF2, HA-Ub and PPAR γ1. Six hours before harvest, a proteasome inhibitor MG 132 (20 µM) was administrated. The result in Fig. 3a left (the middle) shows that MuRF2 interacted with PPAR γ1, and MuRF2 modified PPAR γ1 via poly-ubiquitination. Then, the ubiquitination reaction was assayed and depicted in Fig. 3b. The result indicates that PPAR γ1 proteins were precipitated by anti-His antibody (MuRF2 protein), and the smeared PPAR γ1 K222R protein (lane 2) decreased significantly compared to that of the PPAR γ1 (lane 1) and PPAR γ1 mutants K242R, K68R, K356R, or K228R (lane 3, lane 4, lane 5, lane 6) (Fig. 3b left, the middle). Additionally, the immunoprecipitation of PPAR γ1 by anti-HA (ubiquitin) and the immunoblot of PPAR γ1 indicated that the smeared PPAR γ1 K222R almost disappeared (Fig. 3b left, the bottom, lane 2). Based on above, we believe that the residue K222 is the acceptor of MuRF2 ubiquitinating PPAR γ1. To test the function of K222, the following experiments were conducted.

### MuRF2 weakens the stability of PPAR γ1 protein by ubiquitination modification targeting K222

**Figure 4:**
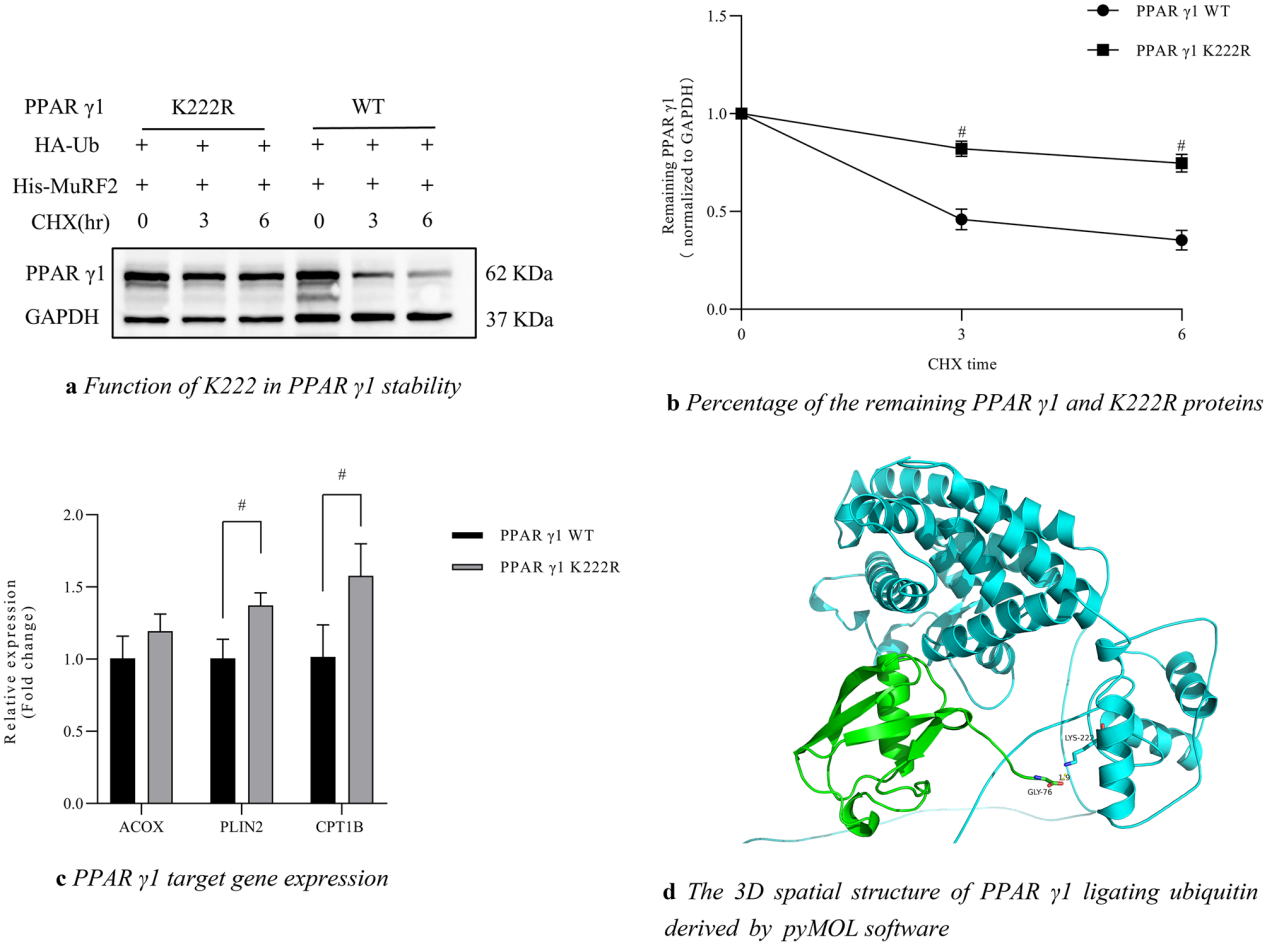
Confirmation of K222 is the key site of MuRF2 ubiquitination modification PPAR γ1 (original blot is presented in Supplementary Fig. S6). (**a**) Function of K222 in PPAR γ1 stability. HEK 293T cells were co-transfected with plasmids His-MuRF2, HA-Ub and PPAR γ1 or K222R mutant. The cells were treated with CHX (final concentration 60 μg/mL) for 3 h and 6 h respectively before harvest. The proteins turnover of PPAR γ1 and PPAR γ1 K222R were determined by immunoblot. Signals of the PPAR γ1 from immunoblots were analyzed using the Image J (National Institutes of Health) and normalized by GAPDH signal. (**b**) Percentage of the remaining PPAR γ1 and K222R proteins. Compared to the significantly weakened PPAR γ1 protein, the remaining K222R proteins remained unchanged after 3 h and 6 h of CHX administration (p < 0.05). (**c**) HEK 293 T cells were transfected with plasmids of His-MuRF2, HA-Ub and PPAR γ1 or the K222R mutant. Samples were subjected to quantitative-PCR analysis of cardiac genes ACOX1, PLIN2 and CPT1b. Data were presented as mean ± SD. n = 3. ^*#*^*p* < 0.05 by two-tailed Student’s *t-*test. (**d**) The 3D spatial structure of PPAR γ1 ligating ubiquitin derived by PyMOL software (version 2.4.0 openvr; DeLano Scientific, San Carlos, CA, USA; https://pymol.org/2/). At position 76 of glycine carboxyl, ubiquitin ligates PPAR γ1 at lysine 222 site. The green is ubiquitin and the blue is PPAR γ1.

Once the ubiquitination site is mutated, ubiquitination reaction is inactivated and the substrate protein always exhibits a longer half-life. To determine whether the PPAR γ1 K222R protein has a longer half-life than protein PPAR γ1, we conducted the CHX chasing experiment. After 48 h of cotransfection of plasmids His-MuRF2, PPAR γ1 and PPAR γ1 K222R, HEK293T cells were treated with CHX for 3 h and 6 h, respectively, and harvested. The result indicated that the protein level of PPAR γ1 K222R was significantly higher than PPAR γ1 as time extends, and PPAR γ1 K222R protein had a longer half-life than PPAR γ1 protein (Fig. 4a, b). The CHX chasing assay verified that K222 is the acceptor of MuRF2 ubiquitination modification PPAR γ1.

### Measurements of PPAR γ1 target genes

To assess the effect of K222 mutation on PPAR γ1 transcriptional activity, the expressions of PPAR γ1-regulated genes ACOX1, PLIN2 and CPT1b, which play roles in cardiac glycolipid metabolism, were measured by RT-qPCR. As shown in Fig. 4c, the mRNA levels of PLIN2 and CPT1b increased dramatically in PPAR γ1 K222R group compared to the PPAR γ1 group (*p* < 0.05), indicating that MuRF2-mediated PPAR γ1 ubiquitination on the residue K222 inhibited PPAR γ1 transcriptional activity selectively.

### The 3D spatial structure of PPAR γ1 ligating ubiquitin derived by pyMOL software

As shown in Fig. 4d, at position 76 of glycine carboxyl, ubiquitin ligates PPAR γ1 at lysine 222 site. The green is ubiquitin and the blue is PPAR γ1. PyMOL software (version 2.4.0 openvr; DeLano Scientific, San Carlos, CA, USA; https://pymol.org/2/) was used to view the graphic.

## Discussion

PPAR γ is a representative member of the ligand-activated nuclear receptor superfamily involved in various critical processes in the development, physiology, reproduction, and homeostasis of organisms. Since its discovery in the early 1990s, PPAR γ has provoked great interest among researchers, and thousands of studies have explored its roles in adipose tissue, liver, colon, heart, endothelium, skeletal muscle, and immune system^1–5,17^. In addition to the originally described PPAR γ1 and PPAR γ2, the other two PPAR γ protein isoforms, PPAR γ1 Δ 5 and PPAR γ2 Δ 5, were recently to be reported to be positively correlated with body mass index in subjects who are overweight and with type 2 diabetic mellitus^18^.

The function of PPAR γ is determined by its expression or activity, and the expression or activity is regulated mostly by posttranslational modifications. Studies have shown that deacetylation at Lys 268 and Lys 293 on PPAR γ can result in increased brown adipocyte genes and energy expenditure, and promote insulin sensitivity^19^. Depending on cellular context and the kinases involved, phosphorylation of PPAR γ S 112 can either decrease or increase PPAR γ activity^20^. Phosphorylation of PPAR γ S 273 by Cdk5 affects series of PPAR γ target genes in obesity^21^. Sumoylation of PPAR γ at Lys107 negatively regulates the target gene transcription^22^; de-sumoylation of PPAR γ K107R in C2C12 myotubes increases the expression of PPAR γ target genes^23^. It has been reported that β-O-linked *N*-acetylglucosamine (O-GlcNAc) modification of PPAR γ1 induces suppression of its trans-activator function^24^. These pathways support that the expression or activity of PPAR γ is mainly depended on the residues and/or the ways of modification. Thus, to investigate the posttranslational modification of PPAR γ further is of great significance for exploring its function.

Ubiquitin-mediated ubiquitination is a ubiquitous posttranslational modification, it is essential for protein turnover and many other cellular functions in eukaryotes^25^. Free ubiquitin is activated and linked to the ubiquitin-activating enzyme (E1) covalently in an ATP-dependent manner; then it is transferred from the E1 and conjugated to the ubiquitin-conjugating enzyme (E2). Finally, ubiquitin ligase enzyme (E3) initiates and assists the transfer of ubiquitin from the E2 to a lysine residue on a substrate molecule. Subsequently, the ubiquitinated substrate is sent to 26S proteasome system and catalyzed, or targeted for change of localization^26–29^. PPAR γ is a lysine-rich protein. The function of PPAR γ ubiquitination in adipocyte differentiation has been illustrated intensively. Studies identified Drosophila seven-in-absentia homolog 2 (SIAH2) and makorin ring finger protein 1 (MKRN1) can ubiquitinate PPAR γ for proteasomal degradation by ubiquitin-dependent pathways and suppressing adipocyte differentiation in 3T3-L1 and C3H10T1/2 cells^30,31^. Neural precursor cell expressed developmentally down-regulated protein 4 (NEDD4), which is an E3 ligase of PPAR γ, stabilizes PPAR γ by inhibiting its ubiquitin-proteasomal degradation; knockdown of NEDD4 in 3T3-L1 adipocytes inhibits the abundance of PPAR γ protein and restrains adipocytes maturation^32^. E3 ligase tripartite motif containing 23 (TRIM23) ubiquitinates PPAR γ2 and leads to reduced proteasomal degradation of PPAR γ2, and subsequently stabilizes PPAR γ2 and promotes adipogenesis in 3T3-L1 cells^33^. Additionally, reports demonstrated that PPAR γ has E3 ubiquitin ligase activity^34,35^, and PPAR γ-mediated ubiquitination and degradation of endoplasmic reticulum membrane selenoproteins S and K are required for adipocyte differentiation^36^. However, ubiquitin-mediated modification of PPAR γ in cardiac tissue and its role is far from clarified. We have previously reported that cardiac specific MuRF2 knockout mice hearts exhibited increased PPAR γ1 protein and deteriorated systolic function in high fat diet induced insulin resistance and cardiomyopathy, indicating the cardioprotection role of MuRF2, and that PPAR γ1 is a potential therapeutic target of diabetic cardiomyopathy; in vitro ubiquitination analyses showed that MuRF2 poly-ubiquitinated PPAR γ1 in a ligand-dependent way and accelerated its proteasomal degradation in HEK293 cells^14^. Another member of the muscle ring finger protein family, MuRF1, is considered highly conserved to MuRF2^37,38^. It was reported that MuRF1 inhibits PPARα activity via monoubiquitination in cardiomyocytes^39^. Despite the fact that deciphering the detailed activity of the ubiquitination process is essential for paving the underlying roles of PPAR α/γ1 in the pathogenesis of diabetic cardiomyopathy, the lysine site(s) that MuRF1/MuRF2 targets for ubiquitinating cardiac PPAR α/γ1 remains unknown.

Identifying ubiquitination sites is critical for whole proteome annotation. High-throughput of mass spectrometry-based proteomics has facilitated numerous ubiquitin-modified proteins and peptides, and has identified vast ubiquitination sites. However, challenging, expensive, and time-consuming given the large number of proteins and proteotypic peptides. Computational tool of prediction is a helpful alternative. This type of tools has gained great popularity with the increasing interest on the identification of ubiquitination sites; thus, many prediction tools have been developed^40–46^. However, most of these tools yield small-scale protein datasets. Recently, tools for large-scale data for predicting ubiquitination sites have been developed to address the limitation. Support vector machine (SVM) software is a widely used machine learning method for classification, regression, and other learning tasks in many areas^47,48^. Based on SVM algorithm, Huang et al. designed the predictor known as UbiSite^41,42^. To construct the predictor, the researchers not only assessed the single features of amino acid composition, amino acid pair composition and evolutionary information, but also incorporated two or more features into a hybrid approach. Independent testing demonstrated an outstanding performance of the UbiSite software with a sensitivity of 85.10%, specificity of 69.69%, and accuracy of 73.69%. Predictor UbiProber was developed for large-scale predictions of both general and species-specific ubiquitination sites^49^. This model integrated the information of key positions and key amino acid residue features by the SVM machine learning approach. Independent testing has demonstrated the model improved the area under the curve (AUC) of the receiver by about 15% in predicting species-specific ubiquitination sites over the existing tools. In the present study, we employed the UbiSite and UbiProber, and screened out five candidate lysine (K) sites in human PPAR γ1: K68, K222, K228, and K242 and K356, which are highly likely to be modified by ubiquitination (Table 1). Due to the likely incidence of false positive associated with the prediction software, the candidates required artificially designed feature selection. In this study, site-directed mutagenesis combined with in vitro ubiquitination analyses, half-life experiment, and RT-qPCR were applied to verify the function of these residues. The GST-pulldown assay indicated that all the ubiquitination levels of the PPAR γ1 mutants decreased in the presence of MuRF2 except the level of the K68R, demonstrating lysine 68 is likely not the target that MuRF2 ubiquitinates PPAR γ1 (Fig. 2c). It was reported that ubiquitination site(s) transfer occurs during the process of in vitro ubiquitination reactions^50,51^, therefore, we conducted a cell transfection study in vivo to confirm the data. As depicted in the Fig. 3, the ubiquitinated PPAR γ1 K222 was greatly weakened by MuRF2, the ubiquitinated levels of PPAR γ1 K242, K68, and K228 and K356 were not affected by MuRF2 (Fig. 3b, the left). Hereby, it is plausible to designate K222 as the key target of MuRF2-mediated ubiquitination. For further confirmation of the crucial site, we conducted CHX chasing assay and found that the mutant PPAR γ1 K222 protein was stabilized (Fig. 4a) and its half-life was extended dramatically compared to the PPAR γ1 protein (Fig. 4b) in the presence of MG132, indicating K222 is indispensable for MuRF2 induced PPAR γ1 ubiquitination and MuRF2 decreases PPAR γ1 protein stability. Finally, we assessed the function of K222 by measuring the expression of PPAR γ1 target genes ACOX1, CPT1b and PLIN2, which are involved in cardiac glycolipid metabolism. As shown in Fig. 4c, mutation of the residue 222 increased the expression of PPAR γ1 downstream genes selectively, consequently promoted PPAR γ1 transcriptional activity.

In conclusion, our study provided further evidence that MuRF2 is an E3 ligase of PPAR γ1, and Lysine 222 might be one of the target sites on PPAR γ1 for ubiquitination by MuRF2. MuRF2 weakens PPAR γ1 protein stability through ubiquitination modification posttranslationally and makes PPAR γ1 transcriptionally inactivated. Due to the highly similar nature of MuRF1 and MuRF2, MuRF1 could be also a PPAR γ1 directed E3 ligase. However, further investigation is necessary to confirm this theory. Our findings established a crucial role of MuRF2 in regulating cardiac PPAR γ1 activity and expression, and may help to develop novel potential therapeutic strategies to ameliorate diabetic cardiomyopathy.

## Methods

### Prediction of ubiquitination site in PPAR γ1

Amino acid sequence of human PPAR γ1 (gene serial number: NM_ 13,871) was obtained from the National Center Biotechnology Information (NCBI) database (https://www.ncbi.nlm.nih.gov/). All lysine sites were labeled. Combined using the support vector machine (SVM) algorithm, the ubiquitin conjugation web resources UbiSite and UbiProber were applied to screen and predict the ubiquitination sites in PPAR γ1. The one with a SVM score > 0.8 in UbiProber or a SVM score > 0.9 in UbiSite was regarded as a candidate site. (Original tables are presented in Supplementary Tables S1, S2).

### Plasmid construction

According to the CDS sequence of MuRF2, ubiquitin, PPAR γ1 and the predicted sites in PPAR γ1, 8 pairs of primers were designed using Premier 5.0 software and synthesized. The coding nucleotide sequence of each predicted lysine was mutated to the sequence of arginine by site-directed mutagenesis and overlapping PCR method. Full-length PCR products of MuRF2, ubiquitin, PPAR γ1 and the mutants were inserted into GV417 (element order: CMV-MCS-IRES-mCherry-SV40-Neomycin) and digested by Nhe I/BamH I or GV146 (element order: CMV-MCS-IRES-EGFP-SV40-Neomycin) and digested by Xho I/EcoR I (Shanghai GK Gene Chemical Technology). The plasmids were converted to the sensitized cells, inoculated to Kanamycin (50 μg/mL) resistant LB medium and incubated for 12 h at 37 °C. The monoclonal colonies were picked out and put into bacterial liquid for amplification. Finally, all plasmids were confirmed by sequencing. The plasmids used as follows: His-MuRF2, HA-Ub, PPAR γ1, PPAR γ1 K68R, PPAR γ1 K222R, PPAR γ1 K228R, PPAR γ1 K242R, PPAR γ1 K356R. (Original tables are presented in Supplementary Tables S3, S4).

### Cell culture

HEK 293T cells (Procell CL-005, Procell Life Science & Technology Co., Ltd) were cultured in DMEM (BI, Cat. #01-052-1ACS) supplemented with 10% fetal bovine serum (FBS) (VivaCell, Cat. #C04001-500) and 1% Penicillin/Streptomycin/L-Glutamate (BI, Cat. #03-031-5B) in a 5% CO_2_ humidified atmosphere at 37 °C.

### PPAR γ1 protein purification

HEK 293 T cells were transfected with plasmids of PPAR γ1 and the mutants respectively using Lipofectamine™ 3000 reagents (ThermoFisher, Cat.# L3000015) by following the manufacture’s instruction. Total 7.5 μL of Lipofectamine™ reagent and 10 μL of P3000™ reagent were used respectively. The amount of plasmid DNA was 5 μg. After 48 h of transfection, the cells were collected and total protein was extracted by cell lysis buffer (Beyotime, Cat.# P0013). One mL of the extracted protein was mixed with 1 mL equilibrium fluid to adjust PH to neutral; then 2 mL of the mixture was mixed with 100 μL of anti-GFP affinity beads 4FF (Smart-Lifesciences, Cat. #SA070001) and incubated by rocking for 2 h and centrifuged 5000 × g for 1 min at 4 °C. After removing the supernatant, 100 μL eluent was added to the centrifuge tube and incubated by rocking for 10 min at 4 °C; then centrifuged 5000 × g for 10 min at 4 °C, the supernatant that contains the purified PPARγ1 protein was retained. Total protein extracted from the transfected groups was used as negative and positive controls. The proteins were denatured for 5 min at 100 °C, resolved on a 10% Bis–Tris gel with SDS-PAGE running buffer (200 V, 40 min) and transferred to PVDF membrane in transfer buffer (400 mA, 40 min) at 4 °C in a wet-blot unit (Bio-Rad). The membrane was blocked by 5% non-fat milk at room temperature for 1 h, then incubated in rabbit anti-PPAR γ1 antibody (Cell Signaling Technology, Cat. #2443, 1:1000) and rabbit anti-GAPDH antibody (BIOSS, Cat. #bs-2188R, 1:5000) overnight at 4 °C. The membrane was washed 3 × 5 min, then incubated in horseradish peroxidase-labeled anti-rabbit secondary antibody (Abbkine, Cat. #A21020, 1:5000) for 1 h at room temperature. The secondary antibody was visualized using a chemiluminescence kit (Genechem, Cat. #KGP1127) and imaged by CHEMI SCOPE 6300 imaging system (Shanghai, China). (Original blots is presented in Supplementary Fig. S1).

### In vitro ubiquitination experiment

The in vitro ubiquitination assay was conducted as we previously described by using an assay kit. Procedure modification was made according to the manufacturer’s instructions. For each reaction system, 50 μg of the purified PPAR γ1 or the mutants’ protein, 2.5 μL human recombinant GST-E1 (50 nM, Boston, Biochem, Cambridge, Cat. #E-306), 5 μL human recombinant UbcH5c/UBE2D3 (2.5 μM, Boston Biochem, Cambridge, Cat. #E2-627), 5 μL human MuRF2 recombinant protein (1 mg, LifeSensors, Cat. #UB305), and 2.5 μL human recombinant ubiquitin (250 μM, Boston Biochem, Cat. #U-100H) were added to 5 μL reaction buffer (50 mM HEPES, pH 7.5) containing 2.5 μL Mg-ATP solution (Boston Biochem, Cat. #B-20) and 0.6 mM DTT. Ultra-pure water was added to make the total reaction volume to 50 μL. The mixture was incubated for 2 h at 37 °C. The reaction was stopped by adding SDS-PAGE loading buffer (Genechem, Cat. #KGP101X), heated 15 min at 100 °C. Then the proteins were resolved on 6% or 10% Bis–Tris gels with SDS-PAGE running buffer (200 V, 40 min), the separated proteins were transferred to PVDF membranes (400 mA) overnight at 4 °C in a wet-blot unit (Bio-Rad). The membranes were blocked by 5% non-fat milk at room temperature for 1 h, then incubated in goat anti-MuRF2 antibody (Abcam, Cat. #Ab4387, 1:500) and rabbit anti-PPAR γ1 antibody (Cell Signaling Technology, Cat. #2443, 1:1000) overnight at 4 °C respectively. After washing in TBST 3 × 5 min, the membranes were subsequently incubated in Rhodamine labeled rabbit anti-sheep IgG antibody (Bioss, Cat. bs-0294R, 1:1000) or horseradish peroxidase-labeled anti-rabbit secondary antibody (Abbkine, Cat. #A21020, 1:5000) for 1 h at room temperature to detect the relevant primary antibody. Secondary antibodies were detected using chemiluminescence kit (Genechem, Cat. #KGP1127) and photographed using the CHEMI SCOPE 6300 imaging system (Shanghai, China). (Original blots are presented in Supplementary Figs. S2, S3).

### In vivo ubiquitination experiment

HEK 293 T cells were co-transfected with plasmids of PPAR γ1 or PPAR γ1 mutant K68R, K222R, K228R, K242R, K356R respectively, HA-ubiquitin and His-MuRF2. The total amount of plasmid DNA was 5 μg. After 48 h of transfection, the cells were treated with proteasome inhibitor MG132 (final concentration 20 μM) for additional 6 h. Then, the cells were harvested and lysed by adding 100 μL lysis buffer (Beyotime, Cat. #P0013). Protein concentration was determined using BCA protein assay kit (Genechem, Cat. #KGPBCA). Total 500 μg protein was mixed with 5 μg mouse monoclonal anti-His antibody (Beyotime, Cat. #AF5060) and agitated gently on a roller shaker for 12 h at 4 °C. Next, protein A/G agarose beads (Absin, Cat. #abs955) were added and incubated by gently roller shaking for 12 h at 4 °C. The mixture was centrifuged 12,000 × g for 1 min at 4 °C, the supernatant was removed. Bound proteins were then eluted by boiling in 1 × SDS loading buffer, resolved on 6% or 10% Bis–Tris gels (200 V, 40 min) and transferred to PVDF membranes at 4 °C (400 mA, 40 min). After 5% non-fat milk blocking, the membranes were incubated with goat anti-MuRF2 antibody (Abcam, Cat. #Ab4387, 1:500), rabbit anti-PPAR γ1 antibody (Cell Signaling Technology, Cat. #2443, 1:1000), rabbit anti-GAPDH antibody (BIOSS, Cat. #bs-2188R, 1:5000), mouse anti-His antibody (Beyotime, Cat. #AF5060, 1:1000), and mouse anti-ubiquitin antibody (ENZO, Cat. #BML-UW9920, 1:1000) overnight at 4 °C separately; then washed in TBST 5 min × 3. Horseradish peroxidase-labeled anti-rabbit (Abbkine, Cat. #A21020, 1:5000) and horseradish peroxidase-labeled anti-mouse secondary antibodies (Beyotime, Cat. #A0216, 1:5000) were used to detect the corresponding primary antibodies. Secondary antibodies were detected using chemiluminescence kit (Genechem, Cat. #KGP1127) and visualized using the CHEMI SCOPE 6300 imaging system (Shanghai, China). (Original blots are presented in Supplementary Figs. S4, S5).

### Protein stability assay and analysis

HEK 293T cells were co-transfected with PPAR γ1 or PPAR γ1 K222R, HA-ubiquitin and His-MuRF2. The amount of plasmid was 5 µg. After 24 h, cells were incubated with Cycloheximide (CHX, final concentration 60 μg/mL) (Beyotime, Cat. #SC0353) for 3 h and 6 h respectively. Then cells were harvested and total protein was extracted and denatured. Protein concentrations were determined by BCA protein assay kit (Genechem, Cat. #KGPBCA). The preparations were separated on a 10% Bis–Tris gel (200 V, 40 min), transferred to PVDF membrane overnight at 4 °C (400 mA). The membrane was blocked in 5% non-fat milk 1 h at room temperature, then incubated with rabbit anti-PPAR γ1 antibody (Cell Signaling Technology, Cat. #2443, 1:1000). GAPDH was used as an internal control (rabbit anti-GAPDH, BIOSS, Cat. #bs-2188R, 1:5000). Horseradish peroxidase-labeled anti-rabbit secondary antibody (Abbkine, Cat. #A21020, 1:5000) was used to detect the primary antibodies. The blotting was imaged using the CHEMI SCOPE 6300 imaging system (Shanghai, China). (Original blot is presented in Supplementary Fig. S6).

### RT-qPCR analysis

Total RNA was isolated using TRIzol reagent according to the manufacturer’s protocols (TIANGEN, Cat. # DP419). c-DNAs were synthesized from total RNA using PrimeScript™ RT Master Mix (Takara, Cat. #RR036A), amplified and analyzed using Green PCR Kit and Real-time PCR. The mRNA levels of PPAR γ1 regulated genes peroxisomal acyl-CoA oxidase 1 (ACOX1), Perilipin2 (PLIN2) and carnitine palmitoyltransferase 1b (CPT1b) were normalized by GAPDH mRNA expression level. Primers as the shows: ACOX1 (forward: CAC AAG TAA ACC AGC GTG TAA A, reverse: GTT CTT AGC CCA CTC AAA CAA G), PLIN2 (forward: TCA ACT CAG ATT GTT GCC AAT G, reverse: TTT GGT GAG TGC ATT TTC TAC G), and CPT1b (forward: AGA AGC ACC AGA ATA TGT ACC G, reverse: GAG AGC TGA CTC CTA GGT ACT T) and GAPDH (forward: ACA CCA TGT ATT CCG GGT CAA T, reverse: TGT GGG CAT CAA TGG ATT TGG). Statistical analyses of RT-PCR were performed using Prism software. Measurements were expressed as mean ± standard deviation (SD). The paired, two-tailed Student’s *t-*test was used to determine the significance between two groups. *p* < 0.05 was regarded as statistically significant.

### Establishment of the spatial structure of PPAR γ1 ligating ubiquitin

To display the spatial structure visually, we derived the 3D PPAR γ1 molecular by PyMOL software (version 2.4.0 openvr; DeLano Scientific, San Carlos, CA, USA; https://pymol.org/2/).

### Ethics approval and consent to participate

The experimental protocol was approved by the Ethics Review Committee of the General Hospital of Ningxia Medical University (approval number 2020-01), and was conducted following the guidelines of the National Institutes of Health, Animal Care and Use Committee.

## Supplementary Information

Supplementary Information.

## Data Availability

The datasets generated during and/or analyzed during the current study are available from the corresponding author upon reasonable request.
