# Supplementary material for: Lysine 222 in PPAR γ1 functions as the key site of MuRF2-mediated ubiquitination modification
Source: Sci Rep. 2023 Feb 3;13:1999. doi: 10.1038/s41598-023-28905-5 (PMC9898238; doi:10.1038/s41598-023-28905-5)
Supplement: Supplementary file 1 — Supplementary Information. [file 41598_2023_28905_MOESM1_ESM.pdf]

Supplementary Materials

Lysine 222 in PPAR γ1 functions as the key site of MuRF2-mediated ubiquitination modification

Yucheng Fan, Fangjing Xu, Rui Wang, Jun He

Supplementary Tables S1 to S4

Supplementary Figures S1 to S6

Supplementary Tables S1

peroxisome proliferator-activated receptor gamma isoform 1 [Homo sapiens]

NCBI Reference Sequence: NP_001361193.2

GenPept Identical Proteins Graphics

```
>NP_001361193.2 peroxisome proliferator-activated receptor gamma isoform 1 [Homo
sapiens]
MVDTEMPFWPTNFGISSVDLSVMEDHSHSFDIKPFTTVDFSSISTPHYEDIPFTRTDPVVADYKYDLKLQ
EYQSAIKVEPASPPYYSEKTQLYNKPHEEPSNSLMAIECRVCGDKASGFHYGVHACEGCKGFFRRTIRLK
LIYDRCDLNCRIHKKSRNKCQYCRFQKCLAVGMSHNAIRFGRMPQAEKEKLLAEISSDIDQLNPESADLR
ALAKHLYDSYIKSFPLTKAKARAILTGKTTDKSPFVIYDMNSLMMGEDKIKFKHITPLQEQSKEVAIRIF
QGCQFRSVEAVQEITEYAKSIPGFVNLDLNDQVTLLKYGVHEIIYTMLASLMNKDGVLISEGQGFMTREF
LKSLRKPFGDFMEPKFEFAVKFNALELDDSDLAIFIAVIILSGDRPGLLNVKPIEDIQDNLLQALELQLK
LNHPESSQLFAKLLQKMTDLRQIVTEHVQLLQVIKKTETDMSLHPLLQEIYKDLY
```

Supplementary Tables S1 Amino acid sequence of human PPAR γ1 (gene serial number: NM_ 13871) was obtained from the National Center Biotechnology Information (NCBI) database (https://www.ncbi.nlm.nih.gov/).

Supplementary Tables S2

A

Ubiquitination sites(SVM score) Confidence: High Low Non

33 (0.2230)	64 (0.5495)	68 (0.6161)	77 (0.6386)	89 (0.4108)
95 (0.3852)	115 (0.2474)	130 (0.3831)	140 (0.3047)	154 (0.4191)
155 (0.3966)	159 (0.4424)	167 (0.4875)	188 (0.6282)	190 (0.7383)
214 (0.8732)	222 (0.9409)	228 (0.9024)	230 (0.8894)	238 (0.8090)
242 (0.8380)	259 (0.6371)	261 (0.6425)	263 (0.6373)	273 (0.7092)
299 (0.6318)	317 (0.4680)	334 (0.7428)	352 (0.8740)	356 (0.8502)
365 (0.7953)	371 (0.5938)	402 (0.2663)	420 (0.6330)	432 (0.5653)
436 (0.7222)	455 (0.5479)	456 (0.5282)	472 (0.5162)	

B

Protein name	Position of site	Flanking residues	SVM Probability
619726	68	RTDPVVADYKYDL–K–LQEYQSAIKVEPA	0.8845
619726	77	KYDLKLQEYQSAI–K–VEPASPPYYSEKT	0.7561
619726	222	LRALAKHLYDSYI–K–SFPLTKAKARAIL	0.8319
619726	238	PLTKAKARAILTG–K–TTDKSPFVIYDMN	0.781
619726	242	AKARAILTGKTTD–K–SPFVIYDMNSLMM	0.866
619726	263	MNSLMMGEDKIKF–K–HITPLQEQSKEVA	0.7918
619726	356	QGFMTREFLKSLR–K–PFGDFMEPKFEFA	0.8197
619726	402	IILSGDRPGLLNV–K–PIEDIQDNLLQAL	0.7333
619726	436	HPESSQLFAKLLQ–K–MTDLRQIVTEHVQ	0.793

Gene names	Ub sites	SVM core	Forecast tool	Nucleotide sequence	Modified sequence
PPARG (NM_138711)	K68	0.6161<br>0.8845	UbiSite<br>UbiProber	CTG**AAA**CTT	CTG**AGA**CTT
PPARG (NM_138711)	K222	0.9409<br>0.8319	UbiSite<br>UbiProber	ATA**AAG**TCC	ATA**AGG**TCC
PPARG (NM_138711)	K228	0.9024	UbiSite	ACC**AAA**GCA	ACC**AGA**GCA
PPARG (NM_138711)	K242	0.8380<br>0.866	UbiSite<br>UbiProber	GAC**AAA**TCA	GAC**AGA**TCA
PPARG (NM_138711)	K356	0.8502<br>0.8179	UbiSite<br>UbiProber	CGA**AAG**CCT	CGA**AGG**CCT

Supplementary Tables S2 All lysine sites were labeled. Combined using the support vector machine (SVM) algorithm, the ubiquitin conjugation web resources UbiSite （A）and UbiProber （B） were applied to screen and predict the ubiquitination sites in PPAR γ1.

Table 1 Based on the ubiquitin conjugation web resources UbiProber and UbiSite combined using the SVM score, the residues K68, K222, K228, and K242 and K356 in PPAR γ1 were screened out and regarded as the candidate ubiquitination sites.

Supplementary Tables S3

Name 1

Synonyms p60

See also sequence in UniParc or sequence clusters in UniRef

Tools ▾ Download Add Highlight ▾ Copy sequence

Length 548

Mass (Da) 60,466

Last updated 2005-10-25 v2

Checksum[i] 2AA2E4D4F5C3E3A1

10	20	30	40	50	60	70	80	90	100	110	120	130	140
MSASLNYKSF	SKEQQTMDNL	EKQLICPICL	EMFTKPVVIL	PCQHNLCRKC	ASDIFQASNP	YLPTRGGTTM	ASGGRFRCPS	CRHEVVLDRH	GVYGLQRNLL	VENIIDIYKQ	ESTRPEKKSD	QPMCEEHEEE	RINIYCLNCE
150	160	170	180	190	200	210	220	230	240	250	260	270	280
VPTCSLCKVF	GAHKDCQVAP	LTHVFQRQKS	ELSDGIAILV	GSNDRVQGVI	SQLEDTCKTI	EECCRKQKQE	LCEKFDYLYG	ILEERKNEMT	QVITRTQEEK	LEHVRALIKK	YSDHLENVSK	LVESGIQFMD	EPEMAVFLQN
290	300	310	320	330	340	350	360	370	380	390	400	410	420
AKTLLKKISE	ASKAFQMEKI	EHGYENMNHF	TVNLNREEKI	IREIDFYRED	EDEEEEEGGE	GEKEGEGEVG	GEAVEVEEVE	NVQTEFPGED	ENPEKASELS	QVELQAAPGA	LPVSSPEPPP	ALPPAADAPV	TQGEVVPTGS
430	440	450	460	470	480	490	500	510	520	530	540		
EQTTESETPV	PAAAETADPL	FYPSWYKGQT	RKATTNPPCT	PGSEGLGQIG	PPGSEDSNVR	KAEVAAAAAS	ERAAVSGKET	SAPAATSQIG	FEAPPLQGQA	AAPASGSGAD	SEPARHIFSF	SWLNSLNE	

Q9BYV6-2

Supplementary Tables S3 We used human p60A isoform (NM_184085) in this study, the length is 548bp and the mass is 60.466 kDa.

Supplementary Tables S4

TGGGAGTTTGTTTTGGCACCAAAATCAACGGGACTTTCCAAAATGTCGTAACAACTCCGCCCCATTGACGCAAATGGGCGGTAGGCGTGTACGGTGGGAGGTCTATATAAGCAGAGC
TGGTTTAGTGAACCGTCAGATCCGCTAGCCGCCACCATGAGCGCATCTCTGAATTACAAATCTTTTTCCAAAGAGCAGCAGACCATGGATAACTTAGAGAAGCAACTCATCTGTCCCA
TCTGCTTAGAGATGTTCACGAAACCTGTGGTGATTCTCCCTTGTCAGCACAACCTGTGTAGGAAATGTGCCAGTGATATTTTCCAGGCCTCTAACCCGTATTTGCCCACAAGAGGAGG
TACCACCATGGCATCAGGGGGCCGATTCCGCTGCCCATCCTGTAGACATGAAGTGGTTTTGGATAGACATGGGGTATATGGACTTCAGAGGAACCTGCTGGTGGAAAATATCATTGAC
ATCTACAAGCAGGAGTCCACCAGGCCAGAAAAGAAATCCGACCAGCCCATGTGCGAGGAACATGAAGAGGAGCGCATCAACATCTACTGTCTGAACTGCGAAGTACCCACCTGCTC
TCTGTGCAAGGTGTTTGGTGCACACAAAGACTGCCAGGTGGCTCCCCTCACTCATGTGTTCCAGAGACAGAAGTCTGAGCTCAGTGATGGCATCGCCATCCTCGTGGGCAGCAACG
ATCGAGTCCAGGGAGTGATCAGCCAGCTGGAAGACACCTGCAAAACTATCGAGGAATGTTGCAGAAAACAGAAACAAGAGCTTTGTGAGAAGTTTGATTACCTGTATGGCATTTTG
GAGGAGAGGAAGAATGAAATGACCCAAGTCATTACCCGAACCCAAGAGGAGAAACTGGAACATGTCCGTGCTCTGATCAAAAAGTATTCTGATCATTTGGAGAACGTCTCAAAGTT
GGTTGAGTCAGGAATTCAGTTTATGGATGAGCCAGAAATGGCAGTGTTTCTGCAGAATGCCAAAACCCTGCTAAAAAAAATCTCGGAAGCATCAAAGGCATTTCAGATGGAGAAAAT
AGAACATGGCTATGAGAACATGAACCACTTCACAGTCAACCTCAATAGAGAAGAAAAGATAATACGTGAAATTGACTTTTACAGAGAAGATGAAGATGAAGAAGAAGAAGAAGGC
GGAGAAGGAGAAAAAGAAGGAGAAGGAGAAGTGGGAGGAGAAGCAGTAGAAGTGGAAGAGGTAGAAAATGTTCAAACAGAGTTTCCAGGAGAAGATGAAAACCCAGAAAAAG
CTTCAGAGCTCTCTCAGGTGGAGCTGCAGGCTGCCCCTGGGGCACTTCCAGTTTCCTCTCCAGAGCCACCTCCAGCCCTGCCACCTGCTGCGGATGCCCCTGTGACACAGGGGGAG
GTTGTACCCACTGGCTCTGAGCAGACCACAGAGTCTGAAACTCCAGTCCCTGCAGCAGCAGAAACTGCGGATCCCTTGTTTTACCCTAGTTGGTATAAAGGCCAAACCCGGAAAGC
CACCACCAACCCACCTTGCACCCCAGGGAGCGAAGGTCTGGGGCAAATAGGGCCTCCAGGTTCTGAGGATTCGAATGTACGGAAGGCAGAAGTGGCAGCAGCCGCAGCGAGTGAG
AGGGCAGCTGTGAGTGGTAAGGAAACTAGTGCACCTGCAGCTACTTCTCAGATTGGATTTGAGGCTCCTCCCCTCCAGGGACAGGCTGCAGCTCCAGCGAGTGGCAGTGGAGCTGA
TTCTGAGCCAGCTCGCCATATCTTCTCCTTTTCCTGGTTGAACTCCCTAAATGAAGGATCACATCATCATCATCATCATTGAGGATCCGCCCCTCTCCCTCCCCCCCCCCTAACGTTACT
GGCCGAAGCCGCTTGGAATAAGGCCGGTGTGCGTTTGTCTATATGTTATTTTCCACCATATTGCCGTCTTTTGGCAATGTGAGGGCCCGGAAACCTGGCCCTGTCTTCTTGACGAGCAT
TCCTAGGGGTCTTTCCCCTCTCGCCAAAGG

Supplementary Tables S4 The MuRF2 sequence has been provided and included in the Supplementary information file.

Supplementary Figures S1

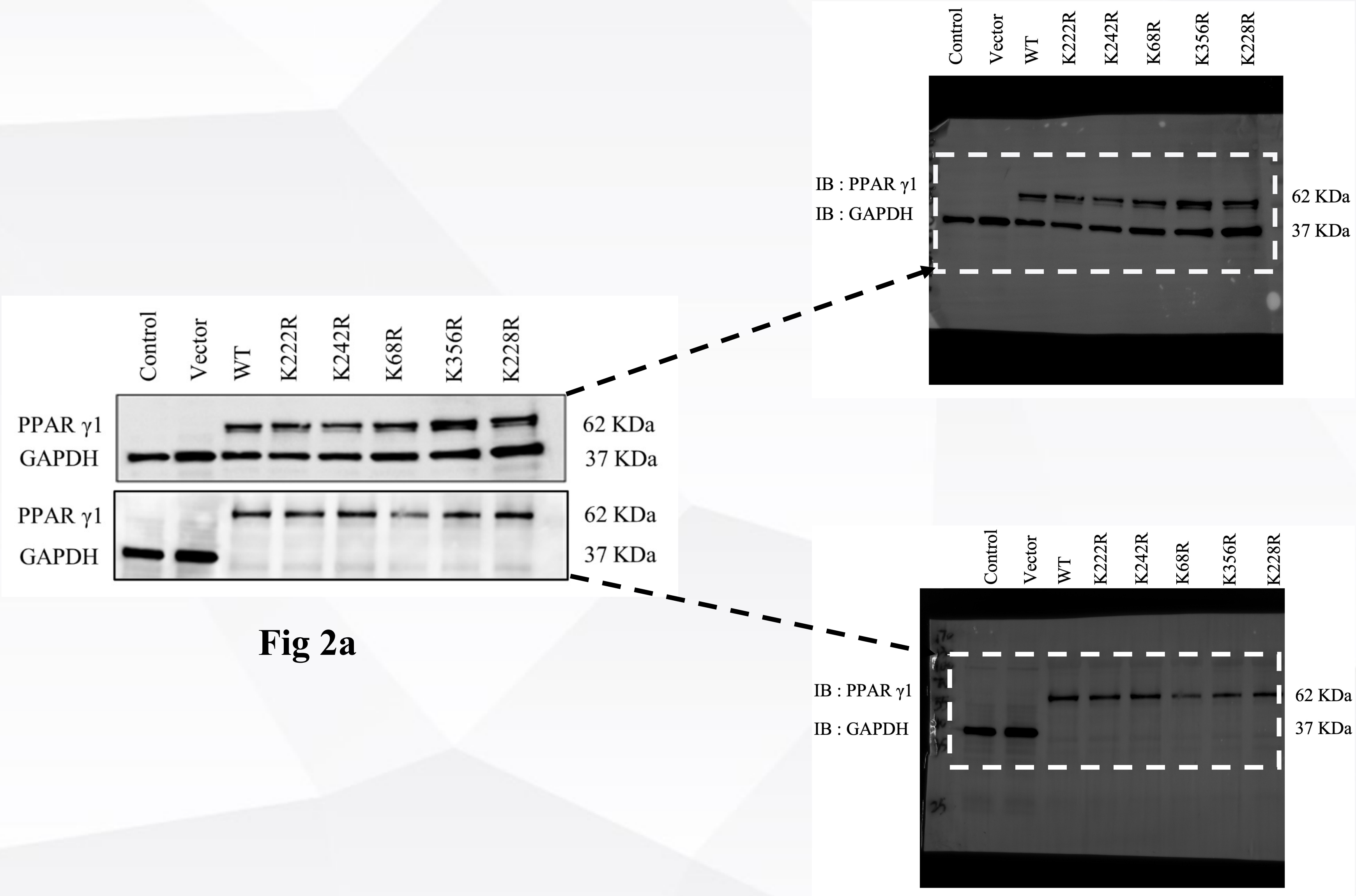

Fig 2a

Figures S1

Supplementary Figures S1

Fig 2a The upper: the over-expression plasmid of PPAR γ1 or the mutant of PPAR γ1 (K68R, K222R, K228R, K242R, K356R) was transfected in HEK293T cells respectively. The PPAR γ1 proteins levels were verified by immunoblot, and no endogenous PPAR γ1 was observed in 293T cells. The lower: immunoblot analysis of the purification efficiency of PPAR γ1 protein. Compared to the negative and positive controls, no GAPDH protein was detected in the total protein derived from the PPAR γ1 and mutants transfected groups, indicating that purified proteins of PPAR γ1 and the mutants were obtained.

Supplementary Figures S2

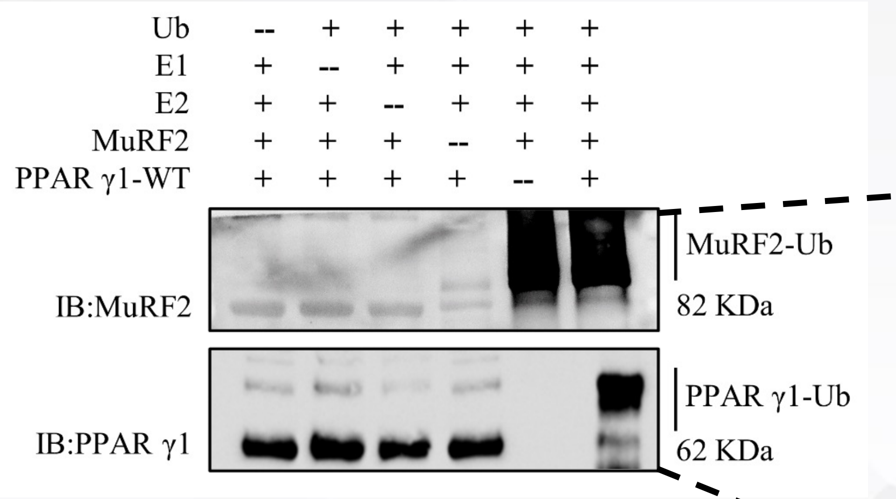

Fig 2b

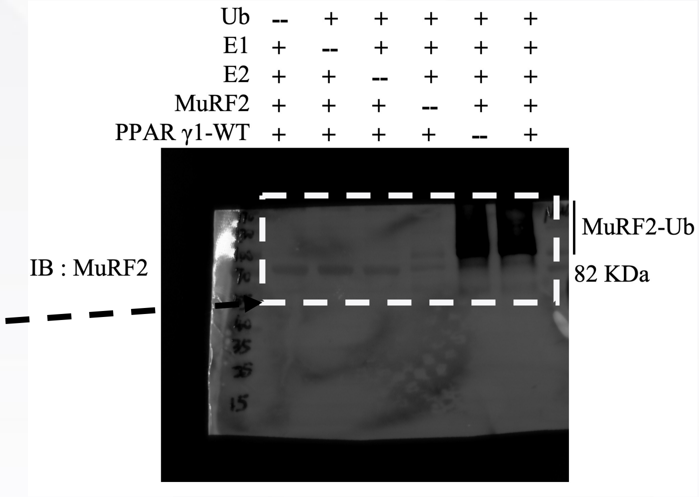

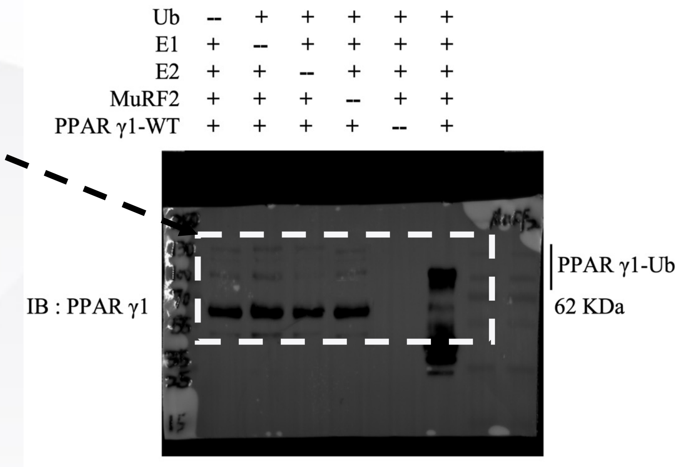

Figures S2

Fig 2b The upper: MuRF2 auto-ubiquitination was demonstrated by immunoblot of MuRF2 (lane 5 and lane 6). The lower: MuRF2's ability to ubiquitinate PPAR γ1. The smeared PPAR γ1 was observed obviously in the full reaction (the far right lane 6).

Supplementary Figures S3

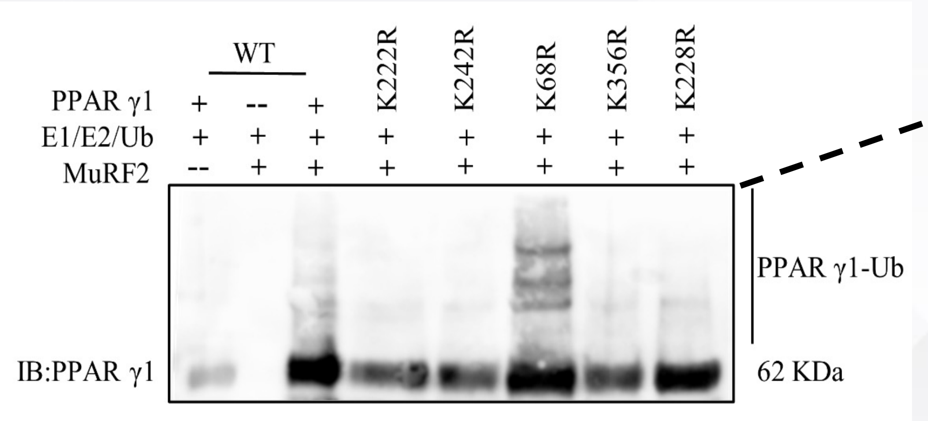

Fig 2c

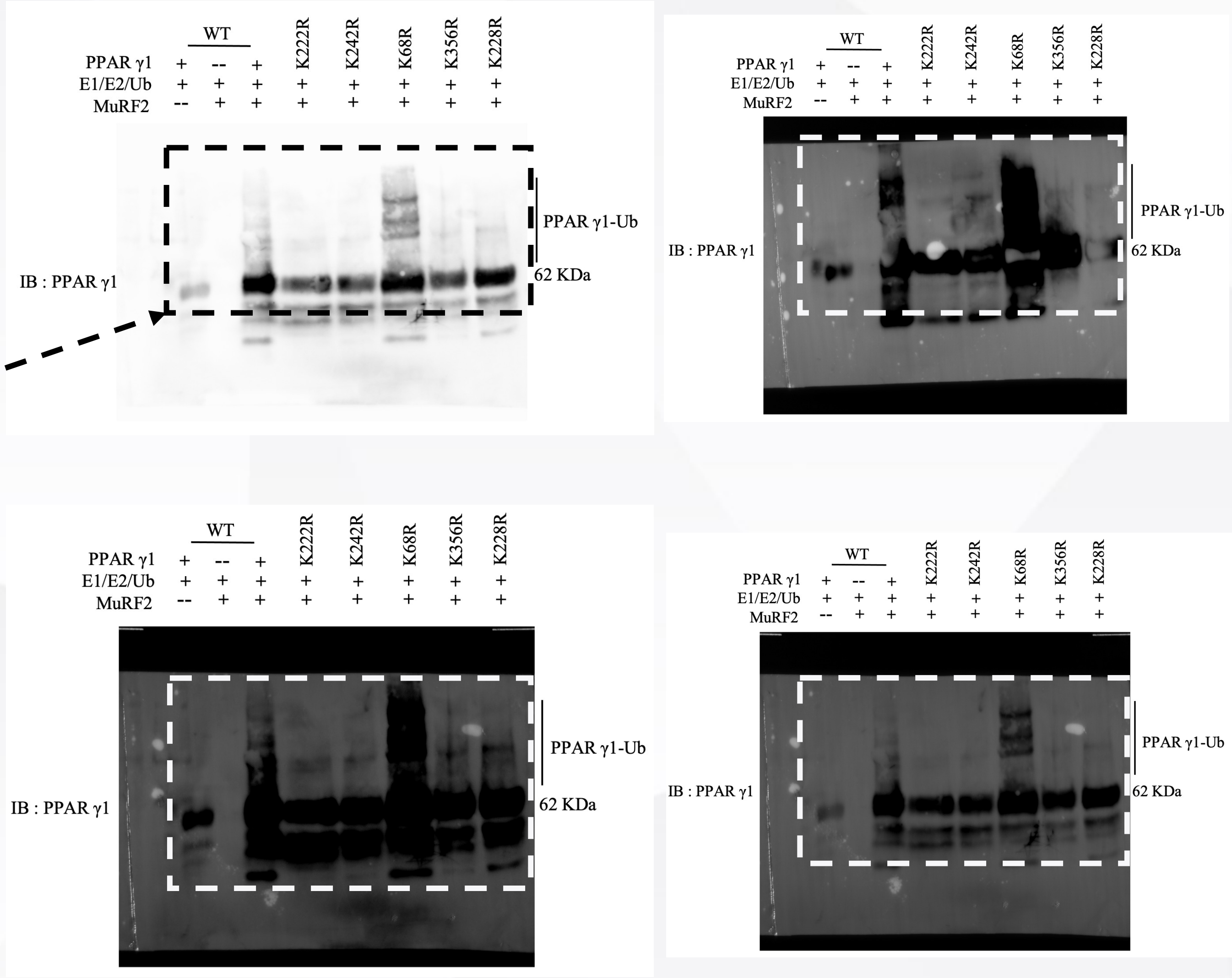

Figures S3

Fig 2c Residue K68 showed the remote possibility of being the ubiquitination site. Except the PPAR γ1 K68R, all the protein stability of the mutant PPAR γ1 K222, K228, and K242 and K356 were weakened in the presence of MuRF2 compared to that of the PPAR γ1 protein, indicating the dispensability of lysine site K68 in MuRF2 mediated PPAR γ1 ubiquitination modification.

Supplementary Figures S4

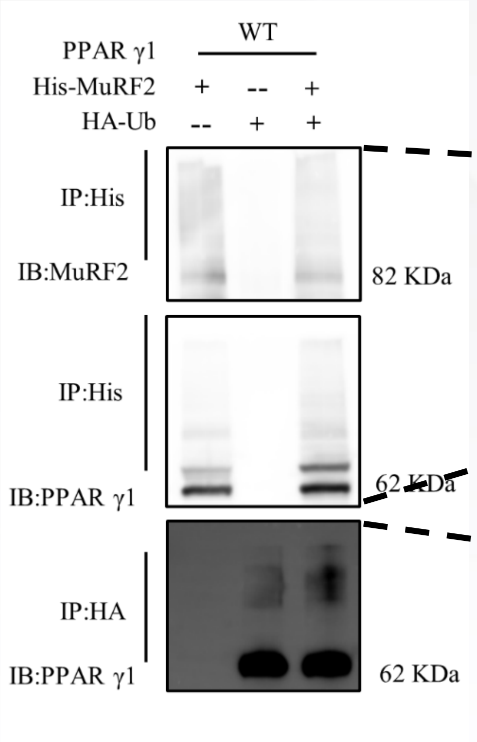

Fig 3a

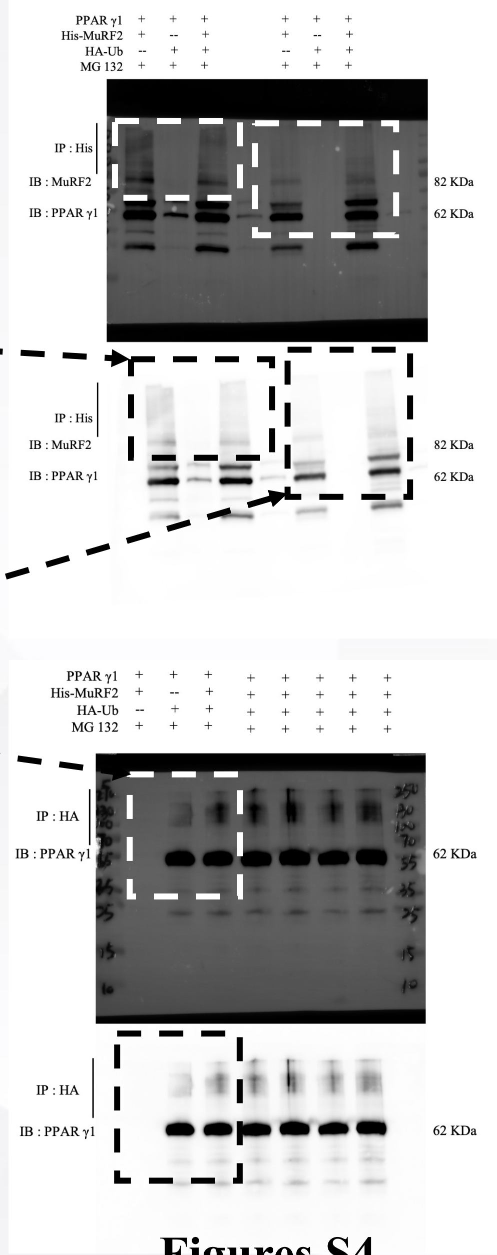

Figures S4

Supplementary Figures S4

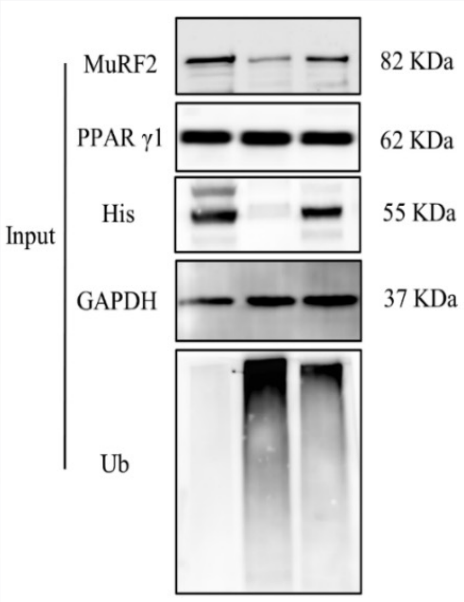

Fig 3a

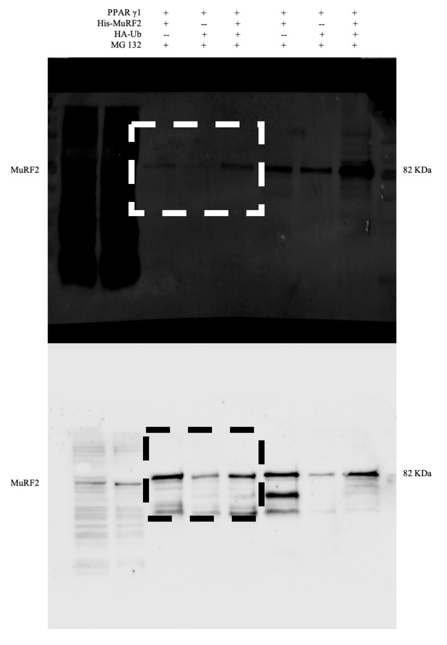

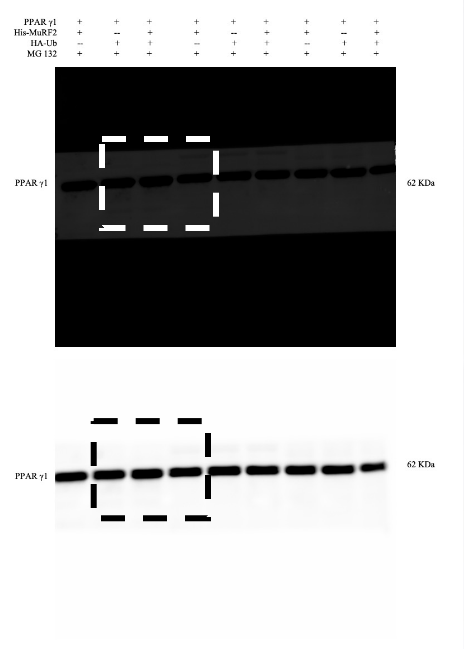

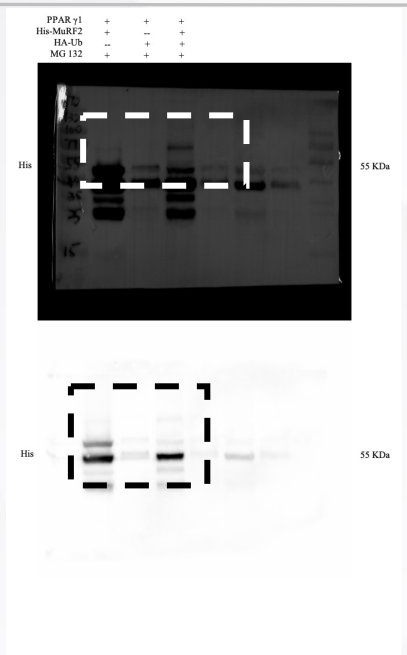

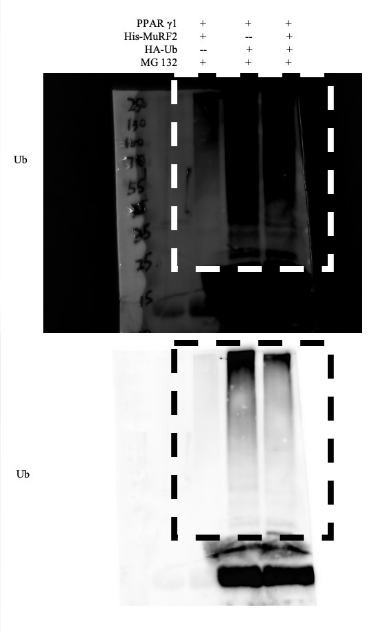

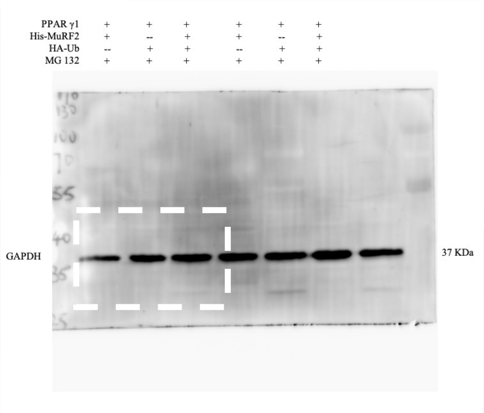

Figures S4

Fig 3a MuRF2 interacted with PPAR γ1 and modified PPAR γ1 via poly-ubiquitination. HEK 293T cells were co-transfected with plasmids His-MuRF2, HA-Ub and PPAR γ1. Cells were treated with proteasome inhibitor MG132 for 6 hours before harvest a, and followed by immunoprecipitation and immunoblot analyses. The immunoprecipitation studies identified the interaction between MuRF2 and PPAR γ1 proteins, and the immunoblots of PPAR γ1 indicated that MuRF2 modified PPAR γ1 protein by poly-ubiquitination (the right lanes of the middle and the bottom).

Supplementary Figures S5

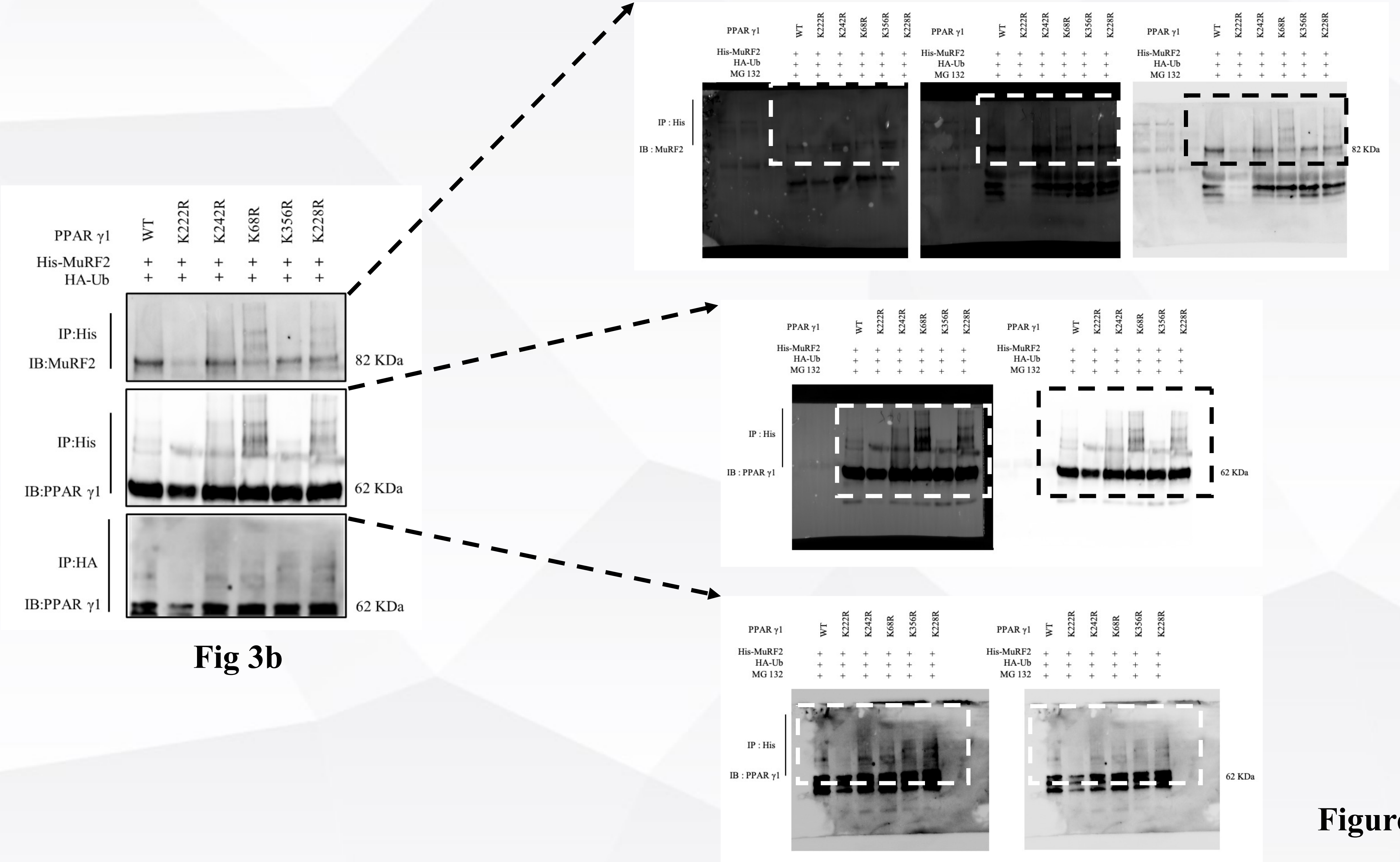

Fig 3b

Figures S5

Supplementary Figures S5

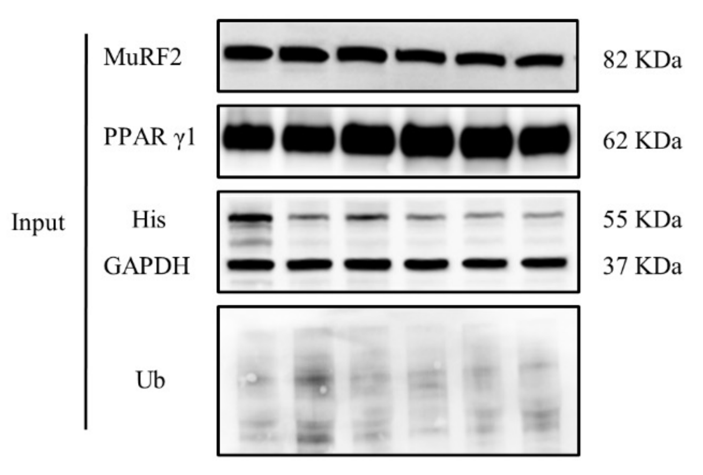

Fig 3b

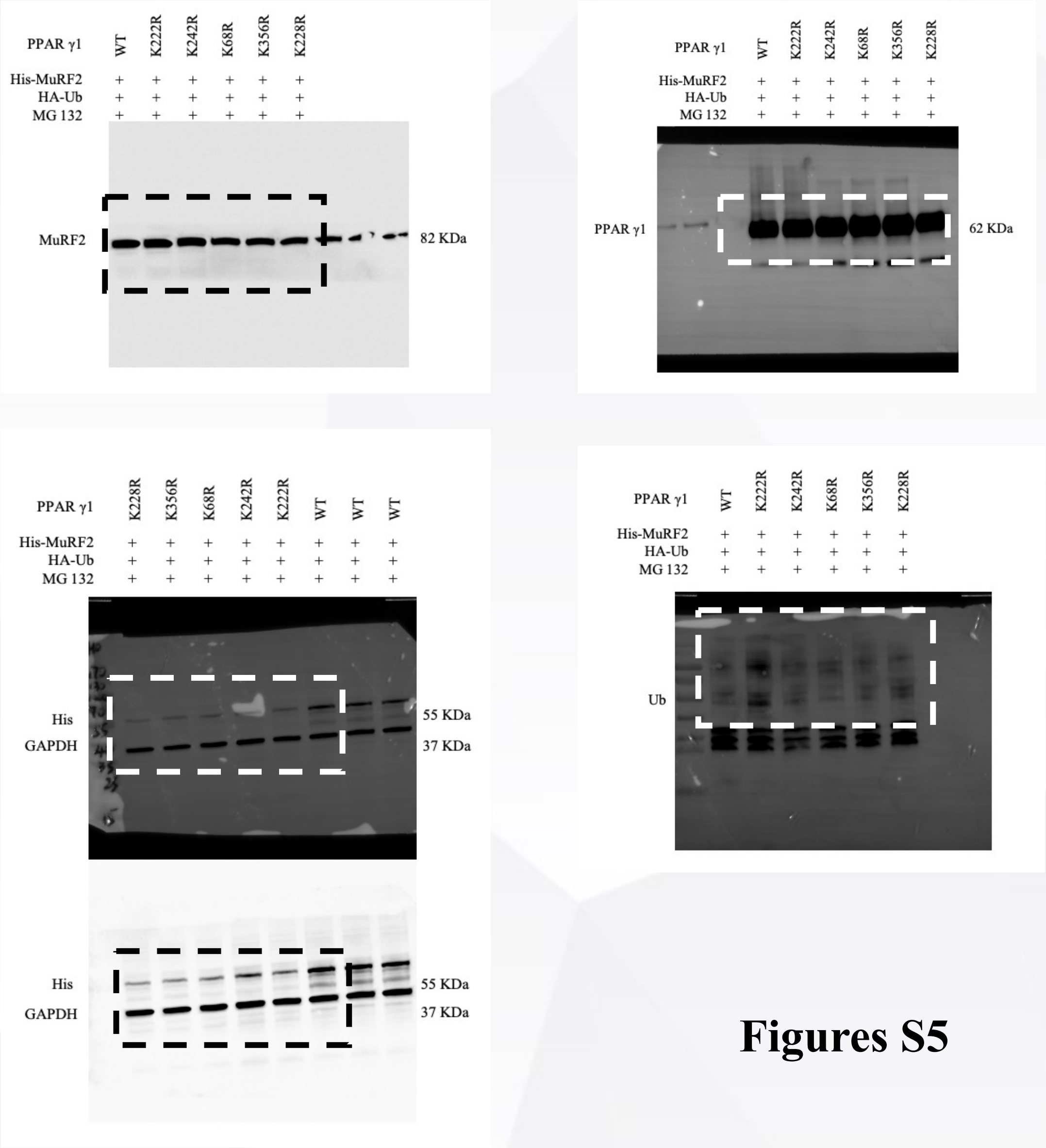

Figures S5

Fig 3b Lysine site 222 exhibited the essential for MuRF2 ubiquitinating PPAR γ1. HEK 293T cells were co-transfected with plasmid of His-MuRF2, HA-Ub and PPAR γ1 wild type or mutant K68R, K222R, K228R, K242R and K356R respectively. The immunoblots of PPAR γ1 demonstrated the ubiquitination level of K222R mutant was significantly reduced (the middle and the bottom of the left figure), indicating residue K222 on PPAR γ1 is the target for MuRF2.

Supplementary Figures S6

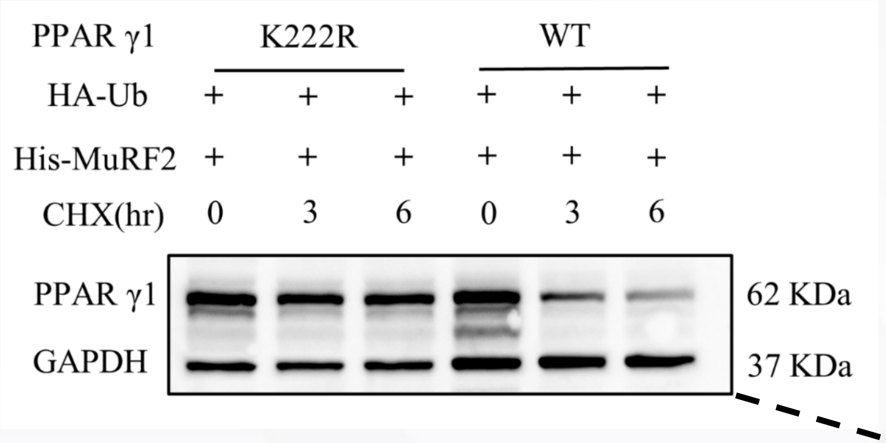

Fig 4a

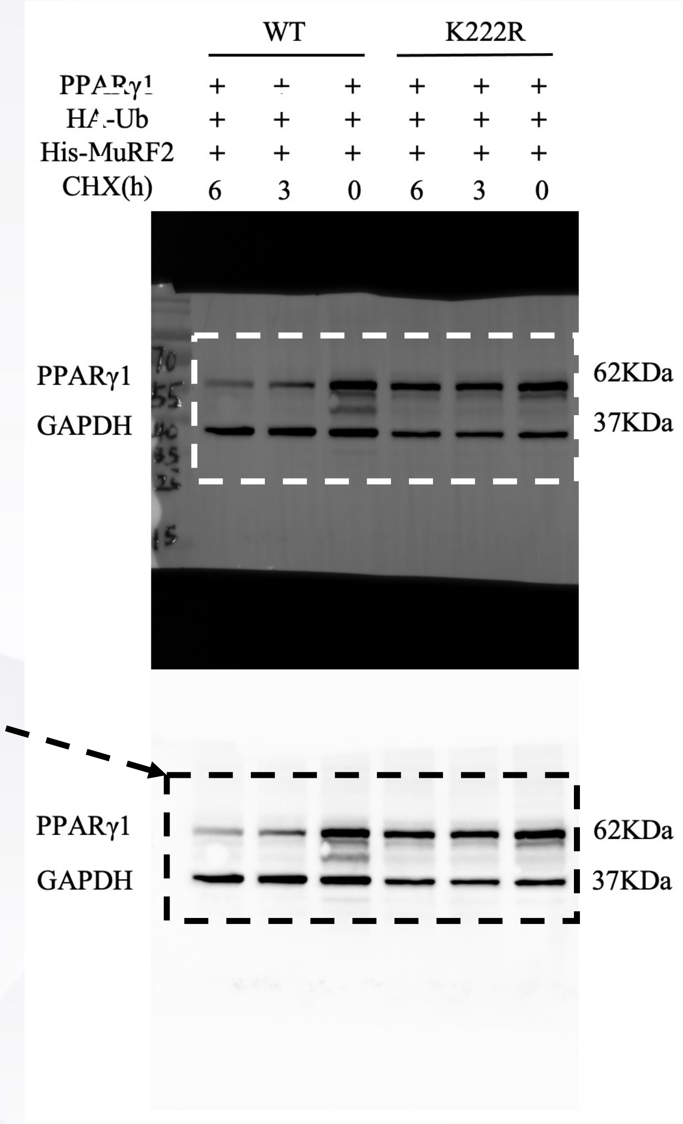

Figures S6

Fig 4a Function of K222 in PPAR γ1 stability. HEK 293T cells were co-transfected with plasmids His-MuRF2, HA-Ub and PPAR γ1 or K222R mutant. The cells were treated with CHX (final concentration 60 μg/mL) for 3h and 6h respectively before harvest. The proteins turnover of PPAR γ1 and PPAR γ1 K222R were determined by immunoblot.
